# CD_8+ _T lymphocytes in bronchoalveolar lavage in idiopathic pulmonary fibrosis

**DOI:** 10.1186/1476-9255-4-14

**Published:** 2007-06-19

**Authors:** Spyros A Papiris, Androniki Kollintza, Marilena Karatza, Effrosyni D Manali, Christina Sotiropoulou, Joseph Milic-Emili, Charis Roussos, Zoe Daniil

**Affiliations:** 1 2^nd ^Pulmonary Department, National and Kapodistrian University of Athens, "Attikon" University Hospital, Athens, Greece; 2 Department of Critical Care and Pulmonary Services, National and Kapodistrian University of Athens, "Evangelismos" Hospital, Athens, Greece; 3 Hematology Department "Evangelismos" Hospital, Athens, Greece; 4 Applied Biomedical Research & Training Laboratory "Marianthi Simou", National and Kapodistrian University of Athens, Greece; 5 Meakins-Cristie Laboratories, McGill University, Montreal, Quebec, Canada; 6 Medical School, University of Thessaly, 41222 Larissa, Greece; 7 Associate Professor of Medicine and Chairman, 2^nd ^Pulmonary Department, National and Kapodistrian University of Athens, Medical School of Athens, "ATTIKON" University Hospital 1 Rimini Street, 12462 Haidari, Athens, Greece

## Abstract

**Background:**

Recently it was shown that in Idiopathic Pulmonary Fibrosis (IPF) tissue infiltrating CD_8+ _T lymphocytes (TLs) are associated with breathlessness and physiological indices of disease severity, as well as that CD_8+ _TLs recovered by bronchoalveolar lavage (BAL) relate to those infiltrating lung tissue. Since BAL is a far less invasive technique than tissue biopsy to study mechanisms in IPF we further investigated the usefulness offered by this means by studying the relationship between BAL macrophages, neutrophils, eosinophils, CD_3+_, CD_4+_, CD_8+_, CD_8+/38+ _TLs and CD_4+_/CD_8+ _ratio with breathlessness and physiological indices.

**Patients and methods:**

27 IPF patients, 63 ± 9 years of age were examined. Cell counts were expressed as percentages of total cells and TLs were evaluated by flow cytometry. FEV_1_, FVC, TLC, RV, *D*LCO, PaO_2_, and PaCO_2 _were measured in all. Breathlessness was assessed by the Medical Research Council (MRC) chronic dyspnoea scale.

**Results:**

CD_8+ _TLs correlated positively (r_s _= 0.46, p = 0.02), while CD_4+_/CD_8+ _ratio negatively (r_s _= -0.54, p = 0.006) with the MRC grade. CD_8+ _TLs correlated negatively with RV (r_s _= -0.50, p = 0.017). CD_8+/38+ _TLs were negatively related to the FEV_1 _and FVC (r_s _= -0.53, p = 0.03 and r_s _= -0.59, p = 0.02, respectively). Neutrophils correlated positively with the MRC grade (r_s _= 0.42, p = 0.03), and negatively with the *D*LCO (r_s _= -0.54, p = 0.005), PaO_2 _(r_s _= -0.44, p = 0.03), and PaCO_2 _(r_s _= -0.52, p = 0.01).

**Conclusion:**

BAL CD_8+ _TLs associations with physiological and clinical indices seem to indicate their implication in IPF pathogenesis, confirming our previous tissue study.

## Background

In idiopathic pulmonary fibrosis (IPF) lung damage leads to defects in mechanics and gas exchange and clinically manifests with breathlessness on exertion[1]. Estimation of breathlessness through the Medical Research Council (MRC) chronic dyspnoea scale is easy to obtain and appears to correlate well with physiological and radiological indices of disease severity and extent in IPF patients [2]. Physiological indices are easily available and highly reproducible measurements providing the physician with information regarding disease severity and extent and more importantly their changes in time are reliable predictors of survival [3,4].

Several studies ascribe a role to the inflammatory cells including neutrophils, macrophages, eosinophils and T lymphocytes (TLs) in the modulation of tissue injury in IPF [5-8]. Recently, we shown that in IPF tissue, infiltrating CD_8+ _TLs are associated with the grade of dyspnoea and physiological indices of disease severity, implicating that they might play a role in its pathogenesis[9], and also that the CD_8+ _TLs recovered by bronchoalveolar lavage (BAL) relate to those in lung tissue[10]. BAL is also of value to study immune and inflammatory mechanisms in IPF[11]. Investigations of tissue and BAL inflammatory cells in IPF have shown that eosinophils, neutrophils and CD_8+ _TLs are associated with tissue fibrosis [6-8,12]. CD_8+ _TLs in particular are associated with a worse prognosis [13]. Since BAL is by far a less invasive technique than tissue biopsy to study pathogenetic mechanisms in IPF we further evaluated the usefulness offered by this means studying the relationship between BAL macrophages, neutrophils, eosinophils, CD_3+_, CD_4+_, CD_8+_, CD_8+/38+ _TLs and CD_4+_/CD_8+ _ratio with breathlessness and physiological indices, in IPF patients.

## Patients and Methods

### Patients

Twenty-seven patients with IPF were included in the study. Seventeen (65%) were male, and the mean (SD) age of all patients was 63 (9) years. Two patients were current smokers and nine were ex-smokers (>20 lifetime-packs of cigarettes but cessation at least 3 months prior to evaluation). They were recruited from the respiratory outpatient clinic of the "Evangelismos" General Hospital, Athens, Greece over a period of 5 years. The diagnosis of UIP/IPF was based on standard criteria [14] which included clinical findings (exertional dyspnoea, non-productive cough, fine bibasilar inspiratory crackles), pulmonary function tests (restrictive pattern and impaired gas exchange), and high-resolution computerized tomography findings (bibasilar honeycombing and reticular abnormalities with minimal ground-glass opacities consistent with the diagnosis of IPF). The diagnosis was confirmed by video-assisted thoracoscopic lung biopsy in sixteen patients. Secondary causes of lung fibrosis were excluded: none of the patients included in this study had a history of environmental or occupational exposure, drug toxicity or connective tissue disease, as documented by patient's history and thorough clinical and immunological work up. Both malignancy and infection were excluded by careful cytology and microbiology examination of BAL fluid in all patients. The study is in compliance with the Helsinki Declaration, was approved by the Institutional Ethics Committee and informed consent was obtained from each patient.

### Pulmonary function tests

The pulmonary function tests included forced expiratory volume in the first second (FEV_1_), forced vital capacity (FVC), FEV_1_/FVC ratio ×100, total lung capacity (TLC), residual volume (RV) and carbon monoxide transfer factor (*D*LCO). TLC and RV were measured by the helium dilution method with a Master Screen apparatus (Erich Jaeger GmbH, Wuerzburg, Germany), and *D*LCO by the single breathholding helium dilution method [15,16]. Lung function measurements were expressed as percentages of predicted values [15,16]. The arterial partial pressure for oxygen (PaO_2_) and carbon dioxide (PaCO_2_) were also measured at rest in all patients.

### Dyspnoea

Dyspnoea was assessed with the modified (6 points) Medical Research Council (MRC) dyspnoea scale score that consists of six questions about perceived breathlessness [17]: category 0, no dyspnoea; category 1, slight degree of dyspnoea (troubled by shortness of breath when hurrying on the level or walking up a slight hill); category 2, moderate degree of dyspnoea (walks slower than people of the same age on the level because of breathlessness); category 3, moderately severe degree of dyspnoea (has to stop because of breathlessness when walking at own pace on the level); category 4, severe degree of dyspnoea (stops for breath after walking about 100 yards or after a few minutes on the level); category 5, very severe degree of dyspnoea (too breathless to leave the house or breathless when dressing or undressing).

### Analysis of BAL cells

All patients underwent fiberoptic bronchoscopy under light sedation before initiation of any kind of corticosteroid or immunosuppressive treatment. The videoscope was wedged to a segmental bronchus of the right middle lobe and lavage was performed using 20-ml aliquots of warmed sterile normal saline (37°C) introduced by syringe through the bronchoscopic aspiration port. A fixed volume of 100–120 ml of saline solution was infused sequentially. The recovered (bronchoalveolar lavage) BAL fluid was obtained through the same syringe and placed on ice. BAL specimens were analyzed within 2 h of being collected. BAL was filtered through nylon sterile gauze to remove mucus, pooled and the total volume was measured. The total cell count was evaluated on an aliquot of the pooled fluid using a Neubauer counting chamber. Cell viability was determined by the trypan blue exclusion test, and in all cases was higher than 90%. The BAL fluid was centrifuged at 400 g, at 4°C, for 10 min. The cell pellet was washed twice with cold phosphate buffered saline (PBS) and resuspended in 4 ml RPMI 1640 medium (Gibco; Grand Island, NY) supplemented with 10% (v/v) heat-inactivated fetal bovine serum (FBS; Gibco; Grand Island, NY). Differential cell counts were made on cytospin preparations. These were made by Shandon cytocentrifuge (Cytospin 3; Shandon Ltd, UK) using 100-μl aliquots of the lavage cell suspensions, adjusted to 4 × 10^5 ^cells/ml. After fixation in methanol, slides were stained with May-Grünwald-Giemsa stain. Differential counts were made from a total count of 400 cells, excluding epithelial cells, and were expressed as a percentage of the total cell count.

Lymphocyte subsets in BAL were evaluated by multiparameter analysis of leukocytes by flow cytometry [18]. Following gentle mixing, 100 μl of 0.5 × 10^6 ^BAL cells were incubated with 10 μl of monoclonal antibody at 4°C for 20 min. For double colour analysis the antibodies were conjugated with fluorescein isothiocyanate (FITC) or phycoerythrin (PE). The antibodies recognizing the following antigens were used in pairs: CD_2_(FITC)/CD_19_(PE), CD_3_(FITC)/CD_4_(PE), CD_3_(FITC)/CD_8_(PE) (Beckman Coulter; France), CD_45_(FITC)/CD_14_(PE), CD_3_(FITC)/CD_16_/CD_56_(PE), and CD_8_(FITC)/CD_38_(PE) (Becton-Dickinson; Belgium). CD_8_/CD_38 _positive cells were identified only in those samples with an adequate number of total cells (16 patients). In each analysis, cells stained by FITC- and PE-conjugated isotype mouse-IgG were used as negative controls. Following incubation the red blood cells were lysed (0.17 M NH_4_Cl lysis buffer) and the stained cells washed with PBS, collected by centifugation and resuspended in 1% paraformaldehyde.

The samples were analyzed using an ELITE ESP flow cytometer (Coulter Electronics; FL, USA), which was equipped with an argon laser providing an excitation wavelength of 488 nm. Before measurement, the optical path was adjusted by testing FlowChek (Beckman Coulter; France). The result of one-half CV was in the range of less than 2%. Data acquisition and analyses were performed with the Elite workstation. A count cycle contained 10000 cells. Using a combination of CD_45_/CD_14 _and light-scatter (FSC/SSC) characteristics, the lymphocytes were identified as small, non-alveolar cells with high CD45 expression.[18] Quadrant markers were set with the isotype control to define the limits of non-specific fluorescence. Measured subpopulations were expressed as percentages of total lymphocytes.

### Statistical Analysis

Data were expressed as means and standard error (SEM). Since BAL cellularity was not normally distributed, correlation coefficients were calculated using non parametric Spearman's correlation coefficient. Furthermore for MRC stepwise ordinal regression analysis was performed to examine the independent effect of inflammatory cells which were found to be significant in correlation analysis. A P-value less than 0.05 was considered statistically significant. Analysis was performed using a SPSS/PC^+ ^program.

## Results

MRC chronic dyspnoea score and lung function data of all patients are listed in Table 1. All patients claimed some degree of dyspnoea (MRC score > 0) and most had a restrictive lung function pattern characterized by a decrease in TLC (mean value less than 65% of predicted) and RV (mean value less than 64% of predicted). The *D*LCO was also decreased in all patients (mean value was less than 50% of predicted).

**Table 1:** Clinical and pulmonary function data* of the study population (n = 27)

MRC score	2.2 ± 0.2 (1–4)
FEV_1 _(% pred)	80.1 ± 2.9 (57–107)
FVC (% pred)	74.7 ± 2.8 (55–99)
FEV_1_/FVC × 100 (ratio)	85.9 ± 1.5 (71–100)
TLC (% pred)	64.5 ± 2.7 (46–96)
RV (% pred)	63.8 ± 5.8 (35–150)
*D*LCO (% pred)	48.9 ± 3 (17–80)
PaO_2 _(mmHg)	73.0 ± 1.95 (53–89)
PaCO_2 _(mmHg)	36.7 ± 0.86 (29–45)

*Values are means ± SEM (with ranges in parentheses).

Table 2 presents the BAL data of the patients. Among the inflammatory cells, lymphocytes appeared more numerous than neutrophils and eosinophils. T lymphocytes (CD_3+ _cells) were the main population of lymphocytes accounting for 74.2% of total lymphocytes. CD_4+ _and CD_8+ _subpopulations shared an almost identical percentage (35.8% and 35.9%, respectively), their ratio being 1.3 ± 0.2.

**Table 2:** Analysis of the differential cell profile in BAL from study population, (n = 27)

Differential cell counts	
Total BAL cell count ×10^3^/ml **of recovered BAL^§^**	134.7 ± 13.6
Macrophages *	82.2 ± 2.7
Neutrophils *	6.8 ± 1.8
Eosinophils *	2.6 ± 0.6
Lymphocytes *	9.1 ± 1.9

**Lymphocyte phenotypes**	

CD_2-_/CD_19+ _cells **(B lymphocytes)	1.9 ± 0.3
CD_3+_/CD_16+_CD_56+_**(cytotoxic lymphocytes)	2.8 ± 0.5
CD_3-_/CD_16+_CD_56+ _**(natural killer cells)	4 ± 0.6
CD_3+ _cells ** (TLs)	74.2 ± 4.6
CD_4+ _cells **(helper TLs)	35.8 ± 3. 9
CD_8+ _cells **(cytotoxic/suppressor TLs)	35.9 ± 3.9
CD_8+/38+ _cells **(activated CD_8+ _cells) (n = 16)	4.0 ± 0.8
CD_4+_/CD_8+ _ratio	1.3 ± 0.2

Values are means ± SEM (with ranges in parentheses). ^§ ^The mean value of recovered BAL is 55.6% with a range of 41.6% to 80% *Values are expressed as percentages of total BAL cell count. ** Values are expressed as percentage of total lymphocytes.

**Figure 1:**
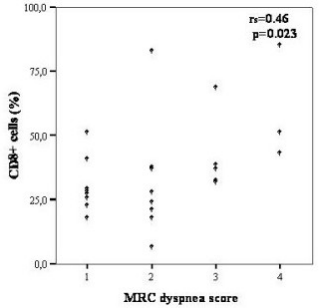
Relationship between BAL CD8+ cells and MRC dyspnea score in IPF patients.

**Figure 2:**
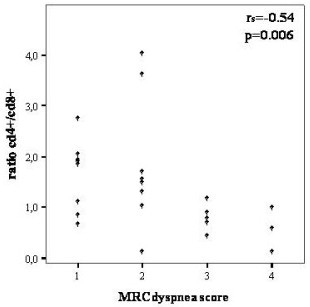
Correlation between CD4+/CD8+ ratio and MRC dyspnea score in IPF patients.

**Figure 3:**
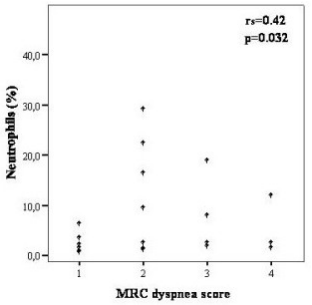
Correlation between percentage of BAL neutrophils and MRC dyspnea score in IPF patients.

Among all inflammatory cells studied, significant correlations with clinical and pulmonary function parameters are shown in Table 3. CD_8+ _TLs showed a positive correlation with the MRC score (r_s _= 0.46, p = 0.02), while the CD_4+_/CD_8+ _ratio correlated inversely (r_s _= -0.54, p = 0.006) [Figures 1 and 2]. A negative correlation was also found between CD_8+ _TLs and RV (r_s _= -0.50, p = 0.017). In the subgroup of patients (n = 16) where the expression of the markers CD_8_/CD_38 _was studied, a negative correlation with FEV_1 _(r_s _= -0.53, p = 0.03) and FVC (r_s _= -0.59 and p = 0.02) was found. The neutrophils showed a positive correlation with MRC dyspnea score (r_s _= 0.42, p = 0.03) [Figure 3], and negative correlation with the *D*LCO (r_s _= -0.54, p = 0.003), PaO_2 _(r_s _= -0.44, p = 0.03), and PaCO_2 _(r_s _= -0.52, p = 0.01). No significant correlations could be identified among the MRC chronic dyspnoea score or lung function parameters and the other cell populations.

**Table 3:** Significant correlations of differential cell counts with clinical and pulmonary function parameters.

	**MRC score**	**RV**	**FEV_1_**	**FVC**	***D*LCO**	**PaO_2_**	**PaCO_2_**
**CD_8+_TLs**	r_s _= 0.46 (p = 0.023)	r_s _= -0.50 (p = 0.017)	-	-	-	-	-
**CD_4+_/CD_8+_TLs**	r_s _= -0.54 (p = 0.006)	-	-	-	-	-	-
**CD_8+_/_38+_TLs**	-	-	r_s _= -0.53 (p = 0.032)	r_s _= -0.59 (p = 0.02)	-	-	-
**Neutrophils**	r_s _= 0.42 (p = 0.032)	-	-	-	r_s _= -0.54 (p = 0.005)	r_s _= -0.44 (p = 0.033)	r_s _= -0.52 (p = 0.01)

The multiple ordinal regression analysis showed that CD_8+ _cells was the only BAL parameter that was significantly related with the MRC dyspnea score, when adjusting for the effect of the neutrophils and the CD_4+_/CD_8+ _ratio. (p = 0.042).

## Discussion

Based on recent studies showing that infiltrating mononuclear cells and especially CD_8+ _TLs could be implicated in the pathogenesis of IPF and the observation that in IPF the TL subpopulations recovered by BAL relate to those in lung tissue [9,10], we further evaluated the relationships between BAL cells and physiologic and clinical parameters of disease severity in IPF patients. Among the different inflammatory cells studied, CD_8+ _TLs correlated positively with the MRC chronic dyspnoea grade and negatively with RV, while the CD_4+_/CD_8+ _ratio correlated negatively with the MRC chronic dyspnea grade. Activated CD_8+/38+ _TLs correlated negatively with the FEV_1 _and FVC. Furthermore, neutrophils correlated positively with the MRC dyspnea grade and negatively with DLCO, PaO_2_, and PaCO_2_.

Associations found in the present study were significant but moderate in comparison to those found between cell subsets in tissue biopsy and the same clinical and physiological parameters [9]. These findings readdress the issue of whether the BAL cellular profile may become a valuable tool to study pathogenetic mechanisms in this group of patients. Furthermore they reinforce the already existing discrepancies on the pathogenetic role of inflammatory cells in IPF patients. Although the current pathogenetic theories in IPF sustain progress despite paucity of interstitial inflammation, the role of inflammatory response in the modulation of tissue injury and fibrosis still remains under investigation [19,20].

In the present study among all inflammatory cells studied in BAL fluid, significant associations were only found for CD_8+ _TLs, activated CD_8+ _TLs and neutrophils implicating some role in the pathogenetic mechanism of IPF. In the present study, the lymphocyte count was found to be less than 12%. Compared with BAL constituents in healthy non-smoker individuals, this value belongs to the normal range of lymphocytosis calculated in normal historical controls [21]. This is in accordance with previous studies in well documented UIP/IPF patients, demonstrating mean lymphocyte counts ranging from 8.5–16.4% in this group of patients [12,22,23]. The clinical value of CD_4+_/CD_8+ _ratio and of CD_8+ _positive lymphocytes exists even in cases with normal lymphocytes count and has been already described both in sarcoidosis and in IPF [12,22]. More precisely, Welker et al demonstrated that even in cases with low percentages of lymphocytes, an elevated CD_4+_/CD_8+ _ratio raises the likelihood for sarcoidosis to more than 85% [22]. As far as IPF patients are concerned, Fireman et al, showed that a lower ratio of CD_4+_/CD_8+ _and a higher number of cytotoxic CD_8+ _cells predicted a worse response to treatment [13]. Therefore subtyping of CD_4+ _and CD_8+ _TLs could be performed even in BAL without severe lymphocytosis.

Regarding CD_8+ _TLs, tissue studies have shown that they infiltrate sites of tissue damage [24], and other studies in BAL have shown that they may also be associated with a worse prognosis [12,13]. In scleroderma lung fibrosis, for instance, CD_8+ _TLs are associated with progressive fibrosis resembling more patients with IPF [25]. Recently we observed that tissue CD_8+ _TLs correlated significantly with physiological and clinical indices of disease severity [9]. The exact mechanisms through which CD_8+ _TLs contribute to lung injury and pulmonary fibrosis are not yet clear. The current hypothesis on the development of IPF conceptualizes ongoing, multiple, small focal episodes of epithelial lung injury followed by a pathologic fibrotic repair mechanism and an imbalance in the expression of T-helper type 1 (Th1) and Th2 cytokines [5,26] CD_8+ _TLs are known to produce type 2 cytokines such as interleukin-4 and interleukin-5 [27]. Recently, it has been hypothesized that in patients with IPF an excessive recruitment of CD_8+ _TLs may occur in response to repeated viral infections and this excessive response may play a role in the development of lung damage through multiple mechanisms (nuclear factor κB, tumor necrosis factor α) of epithelial cells activation, production of chemokines by the alveolar cells which may in turn amplify inflammatory responses in the lung [28]. Furthermore activated CD_8+ _TLs express high levels of CD_38_, a molecule involved in "homing in" of inflammatory cells and cytokine production [29]. Neutrophils, on the other hand, may play a critical role in the induction of lung injury through their capacity to secrete collagenases and other proteolytic enzymes including neutrophils' elastase that degrade different types of collagen and to release oxidants such as superoxide and hydrogen peroxide that damage tissue cells [30].

As far as BAL is concerned, its role in evaluating diffuse parenchymal lung disease remains under investigation. The technique is safe and minimally invasive. In addition lavage samples a much larger area of the lungs that can be obtained by biopsy specimens [11]. In IPF patients it is considered a requirement for the exclusion of other diseases, when biopsy is not performed [14]. Its prognostic importance has been highlighted in interstitial lung disease other than IPF, such as NSIP and scleroderma fibrosis [22,25].

In IPF results are rather controversial. Some studies have shown correlations for at least some of the cell populations studied between BAL and tissue biopsy in the same patients [10,13] and some have not [31]. According to the results of the present study, BAL findings seem to reflect associations between cellular, physiological and clinical parameters developed in previous studies, based on tissue biopsies in well documented groups of UIP/IPF patients [9,12,13,22]. Based on our results, we believe that BAL could be reliably used to assess patients with IPF, not only to exclude infection, malignancy and other interstitial lung diseases but also to unravel the role of inflammatory cells in the pathogenesis of pulmonary fibrosis.

## Conclusion

BAL CD_8+ _TLs associations with physiological and clinical indices seem to indicate their implication in IPF pathogenesis, confirming our previous tissue study.

Based on the non-invasiveness of BAL application, on the quality of information that this tool provides to clinicians on interstitial/alveolar lung milieu and on the recently developing tendency to obviate lung biopsy and to rely more on non invasive methods for diagnosis and follow-up of the disease, [11,32] we conclude that BAL analysis is of importance in evaluating the pathogenetic mechanisms in UIP/IPF patients.

## Abbreviations

**1**. Bronchoalveolar lavage BAL

**2**. Carbon Monoxide Transfer Factor DLCO

**3**. Fluorescein Isothiocyanate FITC

**4**. Idiopathic Pulmonary Fibrosis IPF

**5**. Medical Research Council MRC

**6**. Phycoerythrin PE

**7**. Residual Volume RV

**8**. T lymphocytes TLs

**9**. Total lung capacity TLC

## Competing interests

The author(s) declare that they have no competing interests.

## Authors' contributions

SAP has made substantial contributions to conception and design of the study, has been involved in drafting the manuscript and has given final approval of the version to be published. AK and MK have carried out all BAL specimens' analysis. EDM has been involved in the drafting of the manuscript and critical interpretation of all data. CS performed part of the statistical analysis. JME has made substantial contributions to conception and design of the study and has given final approval of the version to be published. CR contributed in the coordination of all investigators and has given final approval of the version to be published; ZD has contributed in acquisition of all data, in design of the study, in the coordination of all participants and in the final approval of the versions to be published.
